# The NAD-dependent deacetylase SIRT2 regulates T cell differentiation involved in tumor immune response

**DOI:** 10.7150/ijbs.49735

**Published:** 2020-10-03

**Authors:** Cui Jiang, Jingwei Liu, Min Guo, Xiaoxin Gao, Xuan Wu, Ning Bai, Wendong Guo, Na Li, Fei Yi, Rong Cheng, Hongde Xu, Tingting Zhou, Bo Jiang, Tao Sun, Shi Wei, Liu Cao

**Affiliations:** 1 Institute of Translational Medicine, Key Laboratory of Cell Biology of Ministry of Public Health, and Key Laboratory of Medical Cell Biology of Ministry of Education, Liaoning Province Collaborative Innovation Center of Aging Related Disease Diagnosis, Treatment and Prevention, China Medical University, No. 77, Puhe Road, Shenyang North New Area, Shenyang, 110042, Liaoning, China.; 2 Department of Pathology, School of Medicine, University of Alabama at Birmingham, Birmingham, AL 35249-7331, USA.; 3 Department of Medical Oncology, Cancer Hospital of China Medical University, Liaoning Cancer Hospital and & Institute, 44 Xiaoheyan Road, Dadong District, Shenyang 110042, Liaoning, China.; 4 Central laboratory, Cancer Hospital of China Medical University, Liaoning Cancer Hospital and & Institute, 44 Xiaoheyan Road, Dadong District, Shenyang 110042, Liaoning, China.

**Keywords:** SIRT2, T cell, differentiation, breast cancer

## Abstract

Sirtuin 2 (SIRT2), an NAD+-dependent deacetylase, regulates multiple biologic and pathologic processes including mitosis, genomic integrity, cell homeostasis and tumorigenesis. However, the role of SIRT2 in the immune response to cancer remains largely elusive. In this study, we found significantly lower expression of SIRT2 in peripheral T lymphocytes from breast cancer patients when compared to normal individuals. Moreover, SIRT2 levels positively correlated with CD8^+^ effector memory T (T_EM_) cells in breast cancer patients. In keeping with these findings, altered T cells differentiation manifested as decreased T_EM_ cells and increased naive T cells were observed in *Sirt2* deficient mice. The upregulation of CD8^+^ T_EM_ by SIRT2 might attribute to the activation of aerobic oxidation as well as the inhibition of GSK3β acetylation in CD8^+^ T cells. Taken together, these results suggest that SIRT2 participate in tumor immune response by regulating T cell differentiation, which may provide novel insight for tumor prevention and immune therapy.

## Introduction

Sirtuins family members are evolutionarily conserved NAD+-dependent protein deacetylases with diverse subcellular localization and function 1. Seven mammalian sirtuins (SIRT1-7) share a highly conserved core NAD+-binding sequence and a common catalytic domain 2. SIRT2 is the only sirtuin protein mainly localized in the cytoplasm, but has also been reported to exist in the mitochondria and the nucleus 3. Earlier studies have suggested that SIRT2 is a key regulator of multiple biological processes including mitosis, genomic integrity, autophagy, cell homeostasis and differentiation 4. In addition, SIRT2 participates in the regulation of pathologic conditions including multiple nervous diseases, infection and inflammatory reaction 5.

Recent emerging evidence has suggested the potential regulatory role of SIRT2 in carcinogenesis, as *Sirt2*^-/-^ mice develop cancers in multiple organ systems. SIRT2 expression was found to be downregulated in human breast cancers when compared to normal tissues 6. The molecule inhibited proliferation and metastasis of colon cancer cells and demonstrated decreased expression in colon cancer tissues 7. In addition, SIRT2 suppressed proliferation in lung cancer cells and enhances sensitivity to Cisplatin therapy 8. To date, the role of SIRT2 in the immune response against tumor remains largely unknown.

CD8^+^ T cells act as critical regulator of various physiologic and pathologic processes such as infection, autoimmune diseases and cancer 9, 10. In response to tumor, naïve CD4^+^ and CD8^+^ T cells are activated and then differentiate into effector and memory T (T_EM_) cells, of which memory CD8^+^ T cells respond more rapidly than naïve T (T_N_) cells with a more robust response upon repeat stimulation 11, 12. Memory CD8^+^ T cells in circulating blood and lymphoid organs constitute an essential component of long-lived T cell immunity 13. Following tumor infiltration, these resting memory CD8^+^ T cells rapidly exert effector functions and are induced to provide protective immunity against subsequent invasion. CD8^+^ T cell differentiation is modulated by costimulatory signals from antigen presenting cells (APCs) and metabolic pathways as well as epigenetic and transcriptional factors 14-16. Understanding the specific regulatory mechanisms involved in the differentiation of CD8^+^ T cells may provide further insight into antitumor immune therapy.

In the present study, we sought to examine the expression of SIRT2 in T lymphocytes of breast cancer patients and normal individuals. We further investigated the potential molecular mechanisms using *Sirt2* deficient mice. Our findings have suggested that SIRT2 may participate in tumor immune response by regulating T cell differentiation.

## Materials and Methods

### Mice

*Sirt2^-/-^*mice with a deletion of exon 5-8 were a kind gift from Deng CX 6. C57BL/6N wild type mice were purchased from Vital River Laboratory Animal Technology Company as controls. All mice were kept in a specific pathogen-free facility in the Animal Resource Center at China Medical University and were enrolled at 6-8 weeks old. The animal protocols were approved by the Animal Care and Use Committee of China Medical University.

### Patients

The study was approved by the Ethics Committee of Cancer Hospital of China Medical University. A total of 84 breast cancer patients and 24 healthy controls from Cancer Hospital of China Medical University were included in this study. All patients are treatment-naive women who underwent surgery. Patients were consecutively recruited from November 2017 to February 2018. The informed consents were obtained from the patients and healthy controls. The detailed information of all patients and controls were listed in Table 1. The diagnoses of all patients were pathologically confirmed. Whole blood samples (10 mL each) were collected in ethylenediaminetetraacetic acid (EDTA) tubes from healthy donors and patients before surgery.

### Flow cytometry

Human peripheral blood mononuclear cells (PBMCs) were isolated from blood samples through the Ficoll-Paque Plus (17144002, GE) density gradient separation method. The mice spleens were obtained from wild type and* Sirt2*^-/-^mice removing the red blood cells by red blood cell lysis buffer (R1010, Solarbio). Human PBMCs were assessed for live vs. dead status by Zombie NIR dye (423105, Biolegend), while mouse cells were evaluated with PI dye. To detect surface antigens of PBMC, 5×10^6^/cells were washed twice by D-Hanks solution, then suspended in D-Hanks solution and incubated with allophycocyanin (APC), fluorescein isothiocyanate (FITC), phycoerythrin (PE), Brilliant Violet 421 (BV421), PerCP/Cy5.5, Alexa Fluor 647-conjugated monoclonal antibodies on ice for 30 min without light. The antibodies adopted were CD3, CD19, CD4, CD8, CD45RA, CD45RO, CCR7, CD25, CD44, CD62L (BD, Biolegend). The cells stained with surface markers CD4, CD25 were further fixed and permeabilized with IC fixation buffer and permeabilization buffer (00552300, Invitrogen) according to manufacturer instructions, and then incubated with foxp3 antibody or isotype control (Biolegend). CD8^+^ T cells were positively selected with BD FACS Aria II or Sony SH800s cell sorting/analysis instrument. Data were acquired by Sony SH800s, BD FACS Aria II or BD AccuriC6 flow cytometer and analyzed with FCS Express 6 Flow Cytometry RUO Professional software (*De novo* Software; USA) and FlowJo 10.4 (Tree Star; USA).

### Cell culture

The isolated CD8^+^T cells (1×10^6^) were sorted directly into TRIzol reagent (15596026, Invitrogen) and stored at -80 °C prior to RNA extraction. The other sorted cells were cultured at 37 °C in RPMI-1640 medium containing FBS (20%, CLARK, Australia, heat inactivated at 56 °C for 30 min), penicillin (100 U) and streptomycin (100 μg/ml) coated with anti-mouse CD3, clone 145-2C11 (2 ug/10^6^ cells) (100314, Biolegend) and anti-CD28, clone 37.51 (5 ug/10^6^ cells) (102116, Biolegend). SIRT2 specific inhibitor AGK2 (10 μM) with DMSO as control was incubated 24 h for further exploring SIRT2-induced experiments. HEK293T and Jurkat cells were obtained from cell bank of Cao's lab. Cells were cultured at 37 °C in Dulbecco's modified Eagle's medium (DMEM) or RPMI-1640 medium supplemented with 10% FBS.

### Lentiviral production

In order to perform lentiviral production and infection, the control shRNA (shCtrl) lentivirus, shRNA against Sirt2 (shSirt2) and stably express Sirt2 lentivirus were purchased from Shanghai GeneChem Company. The Sirt2 sequence was 5'- CAACCATCTGTCACTACTT -3'; the stably overexpress Sirt2 sequence was 5'- GGAGCCATTTATTGAAACT-3'. Freshly sorted T cells were infected with the lentivirus for at least 60 hours, and the infected efficiency of the target cells was identified by western blot.

### Antibodies and reagents

Antibodies used in this study included SIRT2 (1:1000, S8447, Sigma), GSK3a/β (1:1000, sc-7291, Santa Cruz), GSK3β (1:1000, 12456T, CST), α-tubulin (1:5000, AC012, Abclonal), GAPDH (1:1000, AC012, Abclonal), Flag (1:1000, SG4110-16, Shanghai Genomics Technology) and GFP (1:1000, YM3124, Immunoway). AGK2 (S7577) was purchased from Selleck. DMSO was from Sigma.

### Plasmid constructions and transfection

Human SIRT2 was cloned into pcDNA3.1-flag/HA. Human GFP-GSK3β-isoform1 was purchased from Genechem, China (geneID: 2932, Bank ID: NM_002093). Flag-P300, Flag-CBP and Myc-GCN5 were kindly provided by Qunying Lei (Shanghai Medical College, Shanghai, China). Flag-PCAF was a gift from Weiguo Zhu (Shenzhen University, Shenzhen, China). The plasmids were verified by sequencing and then transfected into HEK293T and MCF-7 cells using lipofectamine 3000 regent (Thermo Fisher Scientific, USA) according to the manufacturer's instructions. Cells were collected 48h after transfection.

### Western blot and Immunoprecipitation

Western blot was performed as previously described 17. For immunoprecipitation, cell lysates were incubated with antibody and Protein A/G-Sepharose beads (sc-2003, Santa Cruz) overnight at 4 °C. The protein-antibody complexes were then washed three times at 4 °C with cold lysis buffer and eluted with SDS loading buffer by boiling for 10 min.

### Quantitative reverse transcriptase polymerase chain reaction (QRT-PCR)

Total RNA was isolated using TRIzol regent, and complementary DNA (cDNA) was synthesized using PrimeScriptII 1st strand cDNA synthesis kit (6210A; TAKARA). QRT-PCR was performed with the Quanti-TectSYBR Green PCR kit (RR820A; TAKARA) using a Roche Light Cycler 480 II sequence detection system. We determined the expression level of Sirt2 in human CD3^+^T cells, and Sirt2, GSK3β and OPA1 in mice CD8^+^T cells. Analyses were performed using the cycle threshold (Ct) method, using the formula 2^-△△Ct^. The following primers were synthesized by Synbio Tech (Suzhou, China).

PCR primary pairs sequences:

- Human Sirt2: forward primer (FP), 5- CTGTCACTACTTCATGCGCCTG-3; and reverse primer (RP) 5- CCTCCACCAAGTCCTCCTGTT-3.
- Human GAPDH: FP, 5- TCAAGGCTGAGAACGGGAAG-3; and RP, 5-TCGCCCCACTTGATTTTGGA-3.
- Mouse Sirt2: FP, 5-CTTCCTTACCCAGAGGCCATC-3; and RP, 5- TCAGCAGGCGGATGAAGTAGT-3.
- Mouse GSK3β: FP, 5-AGAACTGGTTGCCATCAAGAAAG-3; and RP, 5- GAAATACCGCAGTCGGACTATGT-3.
- Mouse OPA1: FP, 5-TGATCTCACCAAGGAGGAAGATC-3; and RP, 5-CCCAGGGCCTTTGACATTT-3.
- Mouse GAPDH: FP, 5- GAGCTGAACGGGAAGCTCAC-3; and RP, 5- TCAGATGCCTGCTTCACCAC-3.

### Measurement of OCR and ECAR

The oxygen consumption rate (OCR) as well as extracellular acidification rate (ECAR) were detected on the basis of Seahorse XFp analyzer (Seahorse Bioscience, 103020-100). 1×10^6^ CD8^+^T cells/well were plated on Seahorse XFp plates for 24 h. The detailed procedure has been previously described 18.

### Statistics

All the statistical analyses were performed using SPSS version 22.0 software (SPSS Inc, Chicago IL, USA) and *p*-values less than 0.05 were considered statistically significant.

## Results

### SIRT2 levels positively correlate with CD8^+^ T_EM_ in breast cancer patients

**Figure 1:**
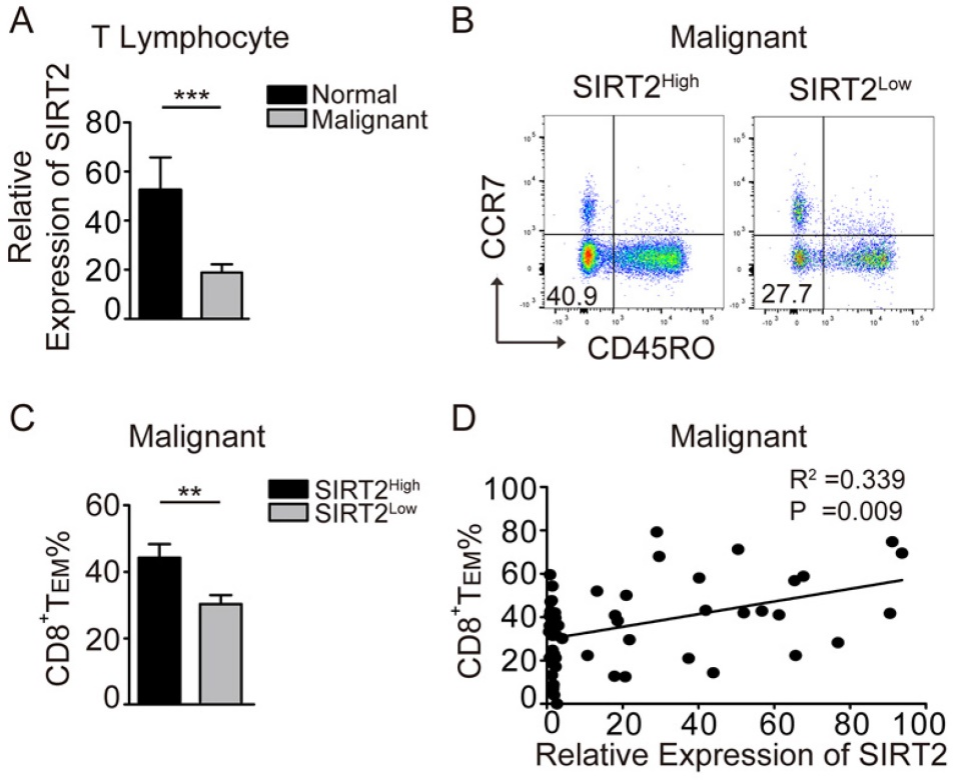
** SIRT2 levels positively correlate with CD8^+^ T_EM_ in breast cancer patients.** A: The expression of SIRT2 was determined by real-time PCR within the T lymphocytes isolated from normal controls (n=19) and breast cancer patients (n=70). B and C: The comparison of CD8^+^T_EM_ (CD45R0^-^CCR7^-^CD8^+^) was carried out in the SIRT2^high^ and SIRT2^low^ groups by flow cytometry. D: The correlation between relative expression of SIRT2 expression and CD8^+^T_EM_ percentage within T lymphocytes was accessed by linear correlation analysis. The data were presented as mean±SD. *P<0.05; **P<0.01; ***P<0.005.

To explore the role of SIRT2 in antitumor immunity, we examined SIRT2 mRNA expression in the peripheral blood CD3^+^ T cells from breast cancer patients and normal individuals. To that end, significantly decreased SIRT2 expression in T lymphocytes was observed in breast cancer patients (Fig. 1A). Furthermore, the patients with SIRT2^high^ T cells had a significantly higher percentage of CD45R0^-^CCR7^-^CD8^+^T_EM_ than those with SIRT2^low^ T cells (Fig. 1B, 1C). Moreover, SIRT2 levels and CD8^+^T_EM_ showed a positive correlation in breast cancer patients (*R^2^*=0.339, *P*=0.009) (Fig. 1D). In contrast, no significant correlation was found between SIRT2 and CD4^+^ T cells (data not shown). The lower SIRT2 expression in T lymphocytes suggested insufficient antitumor immunity in breast cancer patients.

### CD8^+^ T_EM_ cells increase in the peripheral immune system during breast cancer progression

The distribution of immune cells varies in breast cancer with different molecular subtypes and risk levels 19. Therefore, we further analyzed the immune status of breast cancer patients in this cohort. The patients' characteristics were summarized in Table 1. The patients were divided into four groups according to the hormone receptor (HR) ER, PR, HER2 and Ki-67 index: Luminal A (n=17), Luminal B (n=47), HER2-enriched (n=12), and triple-negative (n=8). According to the risk assessment for breast cancer recurrence by 2007 St. Gallen standard, these patients were divided into three groups: low-risk (n=11), intermediate-risk (n=57) and high-risk (n=16).

**Figure 2:**
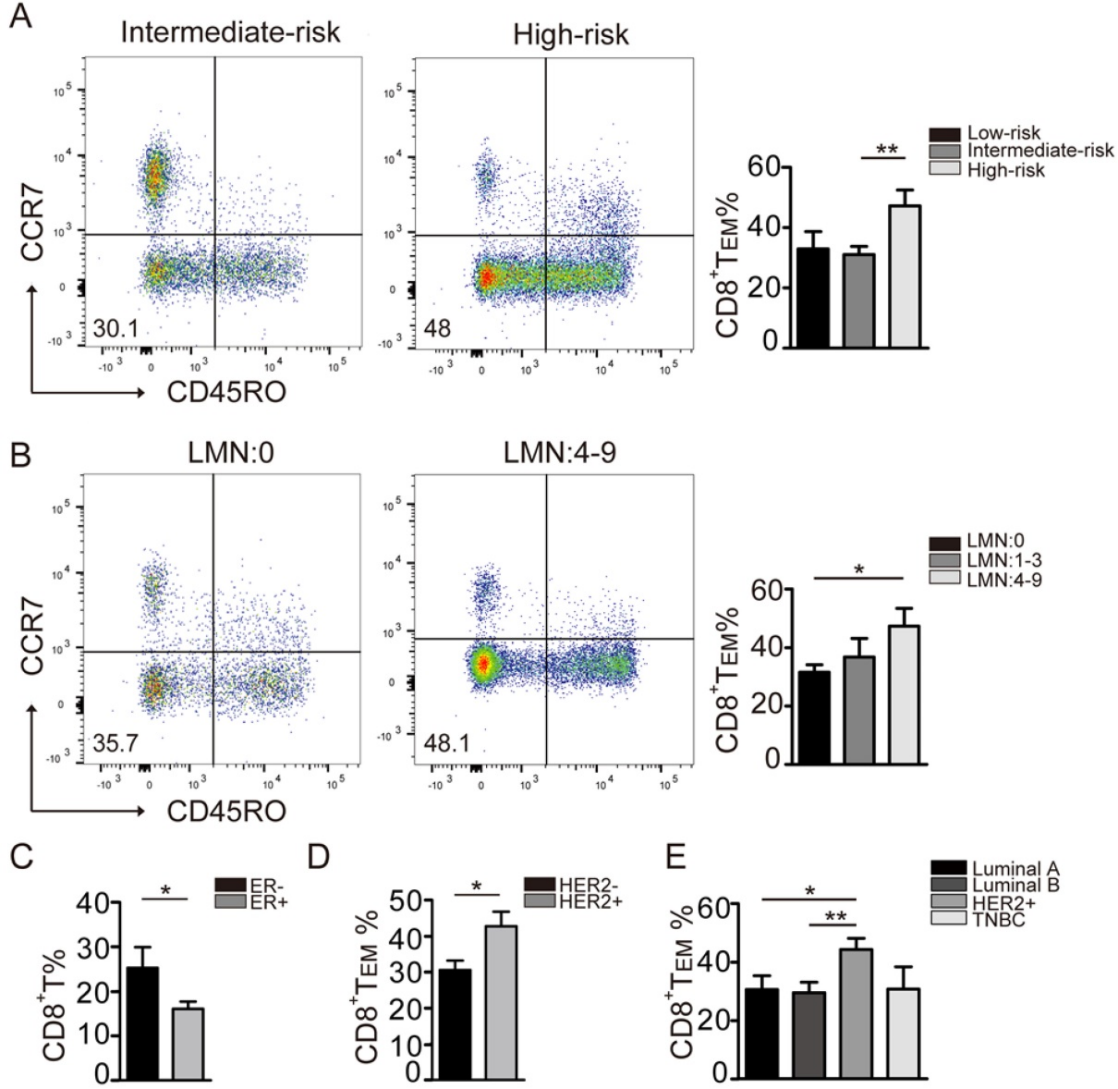
** Distribution of CD8^+^ T cell phenotypes in different breast cancer subtypes.** A: The comparison of CD8^+^T_EM_ (CD45R0^-^CCR7^-^CD8^+^) was performed in different breast cancer recurrence risk groups: Low-risk (n=10), Intermediate-risk (n=47), High-risk (n=12). B: The CD8^+^T_EM_ percentage in pathologic Lymph Nodes metastasis groups: LMN: 0 (n=50), LMN: 1-3 (n=12), LMN: 4-9 (n=8). C: The CD8^+^ T percentage in ER negative (n=17) and ER positive (n=53) groups. D: The CD8^+^T_EM_ percentage in HER2-negative (n=41) and HER2-positive (n=21) groups. E: The CD8^+^T_EM_ percentage in the biologic subtype: Luminal A (n=16), Luminal B (n=26), Her2+ (n=20), TNBC (n=6). The data were presented as mean±SD. *P<0.05; **P<0.01.

To that end, a significantly higher level of CD8^+^ T_EM_ cells was found in the high-risk group when compared to that in the moderate-risk group (Fig. 2A). Furthermore, the levels of CD8^+^ T_EM_ cells positively correlated with lymph node status. Significantly higher levels of CD8^+^ T_EM_ were observed in patients with 4-9 positive lymph nodes when compared to those with no nodal metastasis (Fig. 2B). In addition, CD8^+^ T_EM_ cells were more abundant in patients with ER negative tumors when compared to those with ER positive tumors (Fig. 2C). Moreover, the CD8^+^ T_EM_ cells were significantly increased in the patients with a HER2 subtype tumor (Fig. 2D, 2E). Due to the small number of triple-negative patients enrolled, no obvious trend of CD8^+^ T_EM_ cells was seen in this group. Taken together, these results suggested that CD8^+^ T_EM_ cells were significantly increased in breast cancer subtypes with worse biologic behavior and unfavorable prognosis.

### *Sirt2* deficiency lead to abnormal T cells differentiation

**Figure 3:**
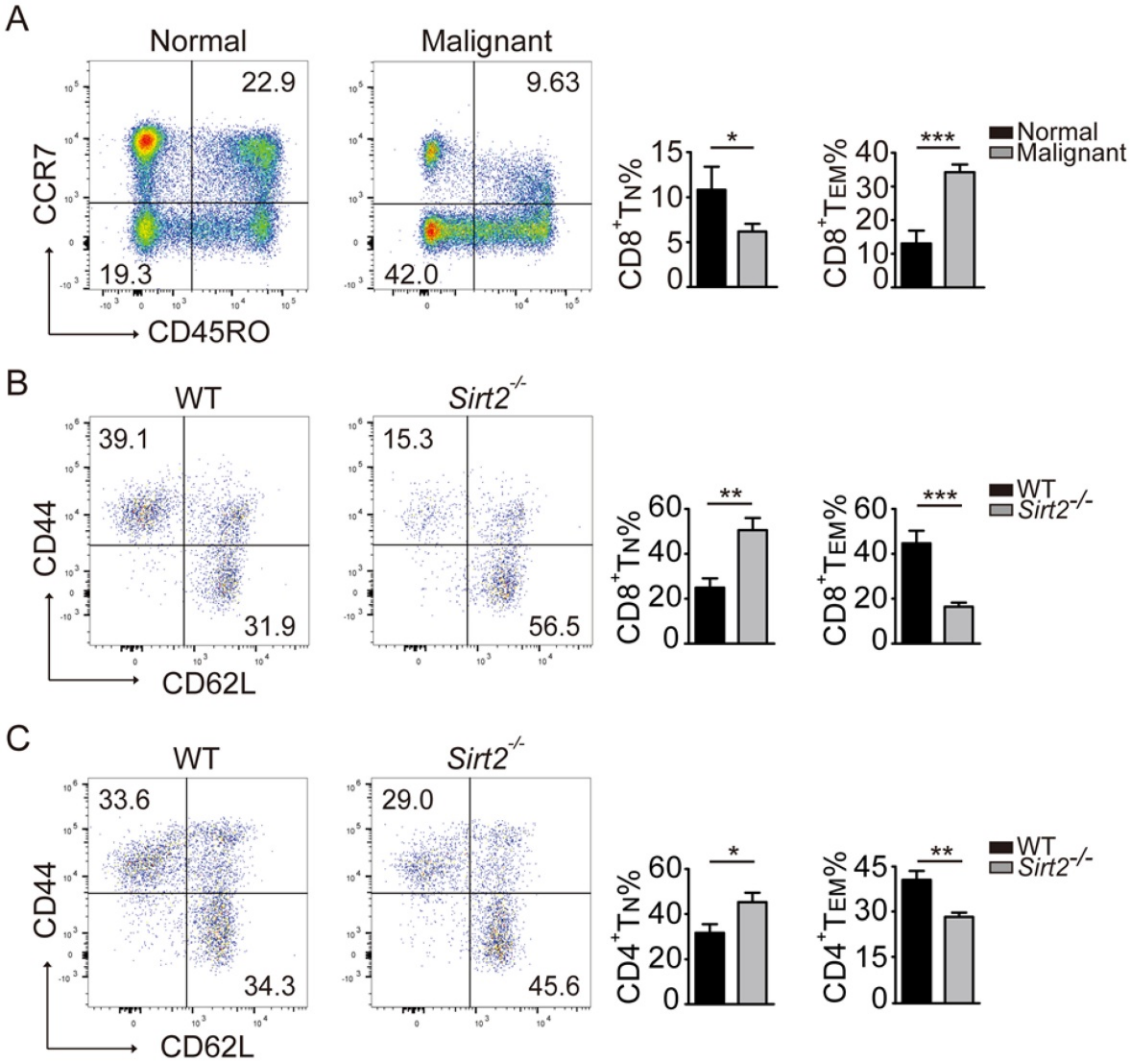
** Distribution of CD4^+^ and CD8^+^ T cells in mice.** A: The CD8^+^ T cell subsets in the normal controls (n=19) and breast cancers (n=70). B.C: The CD8^+^ and CD4^+^ T cell subsets in the homologous mouse: wildtype mice (n=11) and *Sirt2^-/-^* (n=9). The data were presented as mean±SD. *P<0.05; **P<0.01; ***P<0.005.

In response to tumor cells, human naïve CD4 and CD8 T cells were activated and differentiate into effector T cells and memory T cells, and the latter react more rapidly than naïve T cells and provide a more robust response upon repeat stimulation 11. In keeping with this early observation, the subsets of CD45R0^+^CCR7^+^CD8^+^ T_N_ cells in the peripheral blood mononuclear cells (PBMCs) declined in breast cancer patients, with increased proportion of T cells differentiating into CD8^+^ T_EM_ cells (Fig. 3A).

We next used the *Sirt2* knockout mouse model to further investigate the impact of SIRT2 on the immune status. To that end, while there was no significant difference in the distribution of CD4^+^ and CD8^+^T cells (Fig. S2A), consistently increased CD44^-^CD62L^+^ naive T cells (T_N_) and decreased memory T cells (T_M_) in CD4^+^ or CD8^+^ T cells were observed in *Sirt2*^-/-^ mice. Furthermore, this altered differentiation was mainly manifested as a decline in CD44^+^CD62L^-^effector memory T cells (T_EM_) (Fig. 3B, 3C). We did not observe the association between SIRT2 and CD4^+^ T cells in human samples. However, when the mouse *Sirt2* gene was knocked out, SIRT2 also affected CD4^+^ T cells differentiation. Altogether, there results indicate that *Sirt2* deficiency leads to abnormal T cells differentiation including the accumulation of T_N_ cells and decline of T_EM_ cells.

### *Sirt2* deletion restrains aerobic oxidation in CD8^+^ T cells

**Figure 4:**
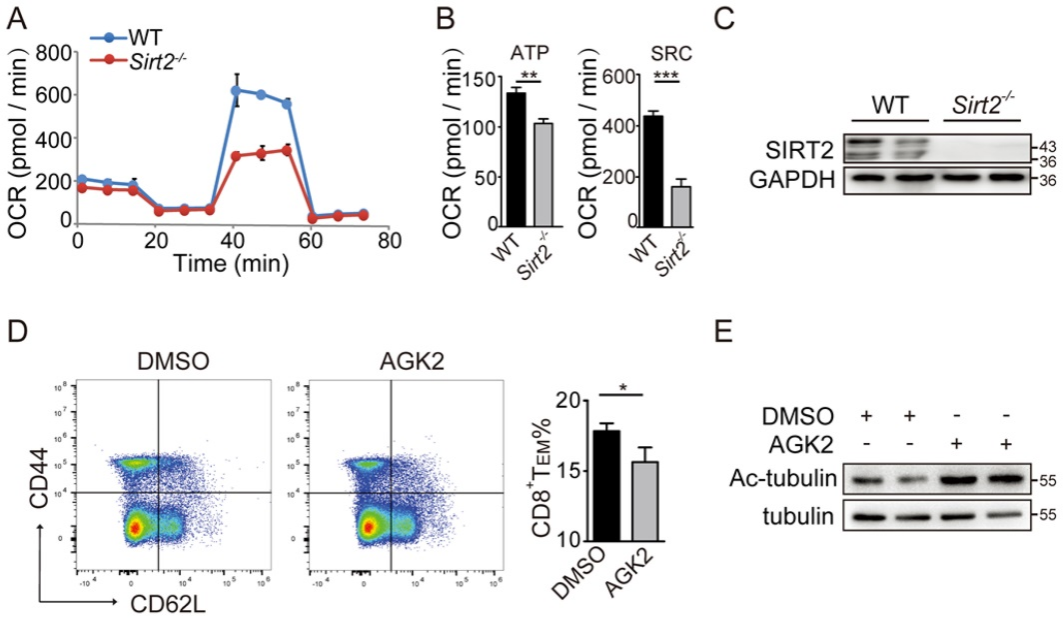
** SIRT2 affects the metabolism of CD8^+^ T cells.** A. C: OCR of wild-type (n=6) and *Sirt2*^-/-^ (n= 6) CD8^+^ T cells in real time after the addition of oligomycin,2,4-dinitrophenol (DNP) and retenone. B: ATP production and spare respiratory capacity of cells. D, E: wild-type CD8^+^ T cells were re-stimulated with anti-CD3 and anti-CD28 for 72 hours and treated with 10 uM/ml AGK2 or DMSO as the control for 24 hours (E). The harvested CD8^+^ T cells were analyzed by flow cytometry (D). The data were presented as mean±SD. *P<0.05; **P<0.01; ***P<0.005.

CD8^+^ T cells, especially the metabolically quiescent T_E_ and T_M_ cells, depend primarily on oxidative phosphorylation to provide sufficient energy for metabolism. Therefore, we sorted the freshly isolated CD8^+^ T cells of *Sirt2*^-/-^ or wild type mice and assessed mitochondrial respiration as well as aerobic glycolysis via detecting the OCR and ECAR in basal conditions or drug-induced mitochondrial stress, to analysis T cells metabolic-flux. For EACR, a small decrease was found in basal glycolysis in *Sirt2*^-/-^ CD8^+^ T cells when compared to the wild type CD8^+^ T cells (Fig. S2B & 2C). For OCR, there was no measurable difference in their basal respiration, leak or non-mitochondrial respiration. Nevertheless, a significant decline in spare respiratory capacity (SRC) in *Sirt2*^-/-^CD8^+^ T cells was detected when compared to wild type CD8^+^ T cells, and a small difference in ATP-coupled was found between the two groups (Fig. 4A-C). In addition, the inner mitochondrial membrane (IMM) fusion protein optic atrophy 1(OPA1), a molecule essential for mitochondrial aerobic oxidation reactions, demonstrated a significant decrease in *Sirt2*^-/-^ CD8^+^ T cells (Fig. S2D & 2E ). Taken together, these finding suggest that SIRT2 regulates CD8^+^ T cell differentiation by altering its aerobic glycolysis.

### SIRT2-mediated CD8^+^ T differentiation relies on glycogen synthase kinase 3β (GSK3β) acetylation

**Figure 5:**
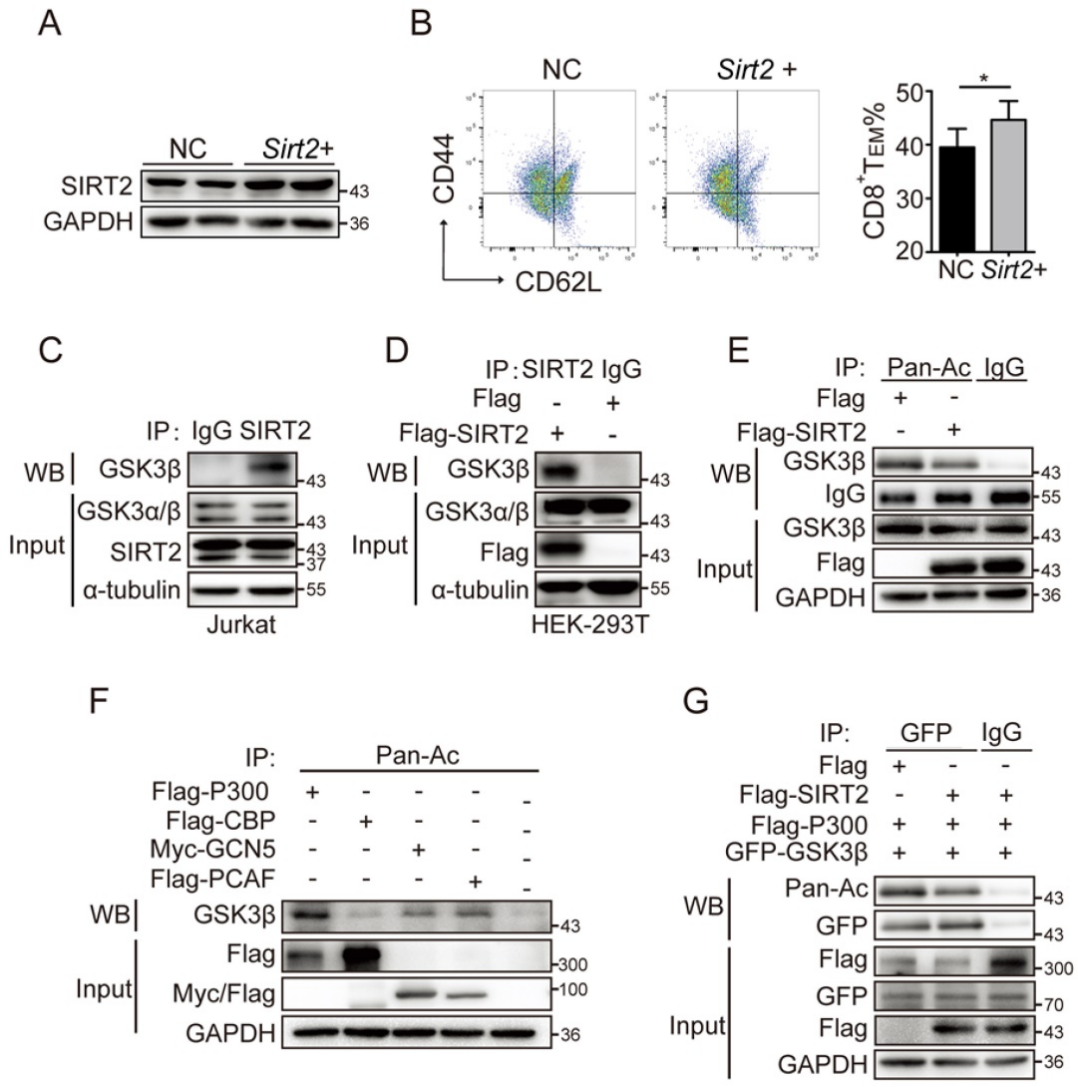
** SIRT2-mediated CD8^+^ T differentiation relies on GSK3β acetylation.** A.B. wild-type CD8^+^ T cells were re-stimulated with anti-CD3 and anti-CD28 for 72 hours and infected with lentivirus overexpressing Sirt2 followed by 12 h activation (A). The harvested CD8^+^ T cells were analyzed by flow cytometry (B). C: SIRT2 interacts with GSK3β *in vivo*. Jurkat cell lysates were subjected to immunoprecipitation with control IgG and anti-GSK3β antibody. The immunoprecipitates were then blotted with the indicated antibodies. D: SIRT2 bind with GSK3β. Flag-tagged SIRT2 was individually transfected into HEK293T cells. The interaction was detected by IP and western blot. E: Catalytic activity of SIRT2 is required for GSK3β deacetylation. Flag-tagged SIRT2 was individually transfected into HEK293T cells. CHK2 acetylation was detected by immunoprecipitation using an anti-acetylated lysine antibody. F: Overexpression of p300, but not other histone acetyltransferases (HATs), could significantly increase GSK3β acetylation. HEK293 cells were transfected into HATs P300, CBP, GCN5 and PCAF. Acetylated GSK3β was purified from cells transfected with Flag-tagged P300, CBP, PCAF or Myc-tagged PCAF. Acetylated GSK3β was determined by immunoblotting. G: SIRT2 overexpression decreases GSK3β acetylation. Flag-tagged GSK3β and P300 was co-transfected with either Flag or Flag-tagged SIRT2 into HEK 293T cells. Acetylation of purified proteins was determined by immunoblotting and western blot. The data were presented as mean±SD. *P<0.05.

Memory cells are enabled to respond more effectively to tumor invasion by multiple mechanisms, including alteration of cytokine receptor expression and epigenetic pathways. Here, we sought to test whether GSK3β was involved in the regulation of SIRT2 on CD8^+^ T cell differentiation. The wild type CD8^+^ T cells in the activation of CD3 and CD28 were cultured for 72 hours and treated with 10 uM/ml AGK2, a selective SIRT2 inhibitor, or DMSO as the control. In AGK2-treated cells, CD8^+^ T_EM_ were slightly decreased when compared to the control group (Fig. 4D & 4E). Furthermore, infecting the cells with lentivirus overexpressing *Sirt2* followed by 12h activation resulted in a higher level of CD8 T_EM_ (Fig. 5A & 5B). Subsequent immunoprecipitation (IP) assay confirmed that GSK3β interacted with SIRT2 in Jurkat cells and HEK-293T cells (Fig. 5C & 5D).

While SIRT2 is a well-known deacetylase, acetylation is also involved in the mechanism of regulating tumor immune responses 20. To assess the acetylation status of GSK3β, HEK293T cells were transiently transfected with Flag-SIRT2, and then assayed for acetylation of GSK3β via IP assay with an anti-acetylated lysine antibody. A significantly decreased acetylated GSK3β was observed when SIRT2 was overexpressed (Fig. 5E). Thus, we next investigated the four classic acetyltransferases to determine which could affect the acetylation of GSK3B. To that end, the ectopic expression of P300, but not CBP, GCN5 and PCAF, significantly enhanced the acetylation of GSK3β, which was verified by Co-IP assay (Fig. 5F & 5G). Together, these results demonstrated that SIRT2 may promote CD8^+^ T cells differentiation by regulating GSK3β acetylation.

## Discussion

**Figure 6:**
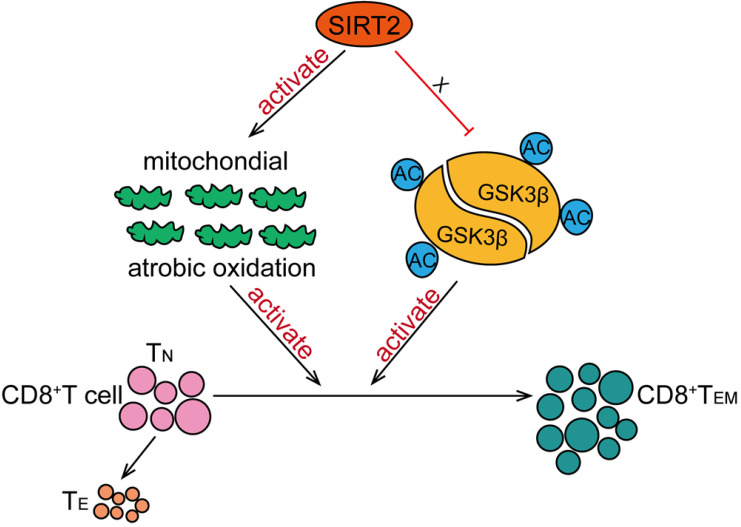
** Schematic model proposed for the role of SIRT2 in T-cell-mediated immune response in breast cancer.** SIRT2 promoted aerobic oxidation, inhibited GSK3β acetylation in CD8^+^ T cells, thus mediating the differentiation of CD8^+^ T cells from T_N_ into T_EM_.

In this study, we have found that SIRT2 participate in T-cell-mediated immune response in breast cancer. SIRT2 levels positively correlated with CD8^+^ T_EM_ cells in breast cancer patients. In addition, *Sirt2* deficiency resulted in abnormal T cells differentiation manifested as declined T_EM_ cells and increased T_N_ cells in mice. Specifically, SIRT2 promoted aerobic oxidation, inhibited GSK3β acetylation in CD8^+^ T cells, thus mediating the differentiation of CD8^+^ T cells from T_N_ into T_EM_ (Fig. 6).

*Sirt2*^-/-^ mice develop gender-specific cancers in multiple organs, which indicate that SIRT2 may function as a tumor suppressor. Indeed, previous studies have reported the disorder of SIRT2 in various types of cancers, suggesting that SIRT2 might suppress tumorigenesis via multiple mechanisms. Here, we discovered previously unrecognized function of SIRT2 in regulating T-cell-mediated immune response: T lymphocytes in breast cancer patients showed decreased SIRT2 expression compared to healthy controls. SIRT2^high^ patients had a significantly higher CD45R0^-^CCR7^-^CD8^+^T_EM_ percentage than the SIRT2^low^ patients. Furthermore, SIRT2 and CD8^+^T_EM_ showed a positive correlation in breast cancer patients. CD8^+^ T cells, including short-lived effector T cells and memory T cells, derive from naive CD8^+^ T cell precursor and supply long-term immunity against tumors 21. The deficiency of T cells and destruction of cytotoxic mechanisms can prompt susceptibility to cancer in animal models 22. In breast cancer, the primary effector immune cells eliminating cancer cells are CD8^+^ CTLs and NK cells 23. Not surprisingly, elevated infiltration of CD8^+^T cells in breast cancer correlated with a favorable clinical outcome 24. In addition, HER2-enrich and basal-like carcinomas are characterized by highest CD8 tumor infiltrating lymphocytes and highest frequencies of memory T cells 25, 26. Researches in both human and mouse models have indicated that CD8^+^ T_EM_ cells are enriched in the bone marrow of breast cancer patients/animals, but deficient in malignant effusions 27-29. During the development of breast cancer, the human immune system is activated, and both CD4^+^ and CD8^+^ T_E_ cells are mobilized to fight tumor cells (Fig. S1). CD8^+^ T cells perform more prominently in the immune response wherein CD8^+^ T_N_ cells became activated, followed by differentiation into cytotoxic effector cells and T_M_ cells, which are simultaneously changed during the progression of the breast cancer (Fig. S1). Altogether, SIRT2 promotes differentiation of CD8^+^ T cells into a memory phenotype. Bulk of CD8^+^ T_EM_ cells are mobilized immediately in immune response and accumulate in more aggressive breast cancer.

We next explored the possible mechanisms by which SIRT2 regulates the immune status in cancer by altering CD8^+^ T cells differentiation. It has been suggested that memory CD8^+^ T cells, known as metabolically quiescent cells, rely primarily on oxidative phosphorylation as their energy source 30, 31. One important feature of CD8^+^ T cell is the upregulation of mitochondrial respiratory capacity, a precondition for the growth and population expansion of CD8^+^ T cells 32, 33. Under mitochondrial stress, CD8^+^ T_EM_ cells demonstrate immediate increase of metabolic characteristics and sustain aerobic glycolysis 30. Therefore, we examined whether differentiation disorder of *Sirt2*^-/-^ CD8^+^ T cells was caused by imbalance of energy metabolism. Using extracted CD8^+^ T cells from SIRT2 knockout mice to detect their aerobic oxidation and glycolysis metabolism, we found a significant decline in SRC and slightly decreased ATP. Importantly, memory CD8^+^ T cells have been suggested to possess an increased SRC and mitochondrial content in CD8^+^ T cells 31. It has been well known that SIRT2 is the only sirtuin mainly located in the cytoplasm. However, recent studies have shown that SIRT2 could also be localized to mitochondria and interact with mitochondrial candidate proteins to direct mitochondrial metabolism 34. In the present study, we found that OPA1, a protein essential for the fusion events of the inner mitochondrial membrane during CD8^+^memory T cells metabolic reactions 10, 35, was also decreased in *Sirt2*^-/-^ CD8^+^ T cells. Therefore, SIRT2 deficiency leads to an energy-deprivation as well as dysfunction in activating CD8^+^ T cells and thus a significant decrease of effective CD8 T_EM_ cells. Our findings suggest that SIRT2 may functions as a critical energetic regulator of CD8^+^ T cells metabolism and differentiation, particularly through aerobic oxidation.

Another potential mechanism is that SIRT2 induces enrichment of CD8^+^ T_EM_ in PBMCs by affecting key signaling pathways of tumor immune system. Among multiple and complex signaling pathways of T cell differentiation, several core molecules regulating memory T cell differentiation were verified and well-characterized 36-39. It has been reported that AKT and its direct substrate GSK3β are critical regulators of CD8^+^memory T cells differentiation 40-42. Several reports indicate that GSK3β is involved in various signaling pathways controlling T cells differentiation, metabolism, cell death and survival 43, 44. Expression of constitutively active GSK3β decreases proliferation of CD8^+^ T cells 39, 45. Additionally, AKT/GSK3β signal pathway activation occurs at mitochondria-endoplasmic reticulum contact sites, which is critical for early mitochondrial reprogramming of memory CD8^+^T cells 33. Here, our findings suggest that SIRT2 induce the deacetylation of GSK3β. The interaction of GSK3β with SIRT2 has been confirmed by IP assay. Therefore, SIRT2 may mediate CD8^+^ T differentiation through the inhibition of GSK3β acetylation. However, more specific and detailed impact of SIRT2 protein on the functionality of antitumor immunity requires further investigation.

## Conclusion

In summary, we have identified an association of SIRT2 with enhanced CD8^+^ T_EM_ cell differentiation in breast cancer, an event mediated at least in part by activated aerobic oxidation as well as the inhibition of GSK3β acetylation in CD8^+^ T cells. These findings provide potential insight into the full biological repertoire of SIRT2 in the immune response against cancer thus warrant further investigation.

## Supplementary Material

Supplementary figures and tables.

## Figures and Tables

**Table 1:** Patient characteristics in the study

		N	%	variable		N	%
**Normal controls**							
**Age**	≤35y	6	25	**Age**	>35y	18	75
**Breast cancer patients**							
**Age**	≤35y	4	4.8	**PR**	-	29	34.5
	>35y	80	95.2		+	55	65.5
**Tumor size**	T1:≤2cm	55	65.5	**HER2**	-	58	33.3
	T2:>2cm≤5cm	26	31		+	26	66.7
	T3:>5cm	3	3.5	**Ki-67**	≤14%	25	29.8
**Lymphatic** **metastasis**	N0:0	56	66.7		>14%	59	70.2
	N1:1-3	17	20.2		Luminal A	17	20.2
	N2:4-9	11	13.1	**Molecular**	Luminal B	47	56
**Stage**	I	36	42.8	**subtype**	HER2+	12	14.3
	II	32	38.1		Basal-like	8	9.5
	III	16	19.1	**Dangerous degree classification**	low-risk	11	29.5
**ER**	-	22	26.2		intermediate-risk	57	67.9
	+	62	73.8		high-risk	16	2.6

Abbreviation: TNBC: triple-negative breast cancer.

## Abbreviations

SIRT2: Sirtuin 2
effector memory T: T_EM_
TN: naive T
APCs: antigen presenting cells
QRT-PCR: quantitative reverse transcriptase polymerase chain reaction
OCR: oxygen consumption rate
ECAR: extracellular acidification rate
HR: hormone receptor
SRC: spare respiratory capacity
IMM: inner mitochondrial membrane
OPA1: optic atrophy 1
GSK3β: glycogen synthase kinase 3β
IP: immunoprecipitation
