# Supplementary material for: The NAD-dependent deacetylase SIRT2 regulates T cell differentiation involved in tumor immune response
Source: Int J Biol Sci. 2020 Oct 3;16(15):3075–84. doi: 10.7150/ijbs.49735 (PMC7545715; doi:10.7150/ijbs.49735)
Supplement: Supplementary file 1 — Supplementary figures and tables. [file ijbsv16p3075s1.pdf]

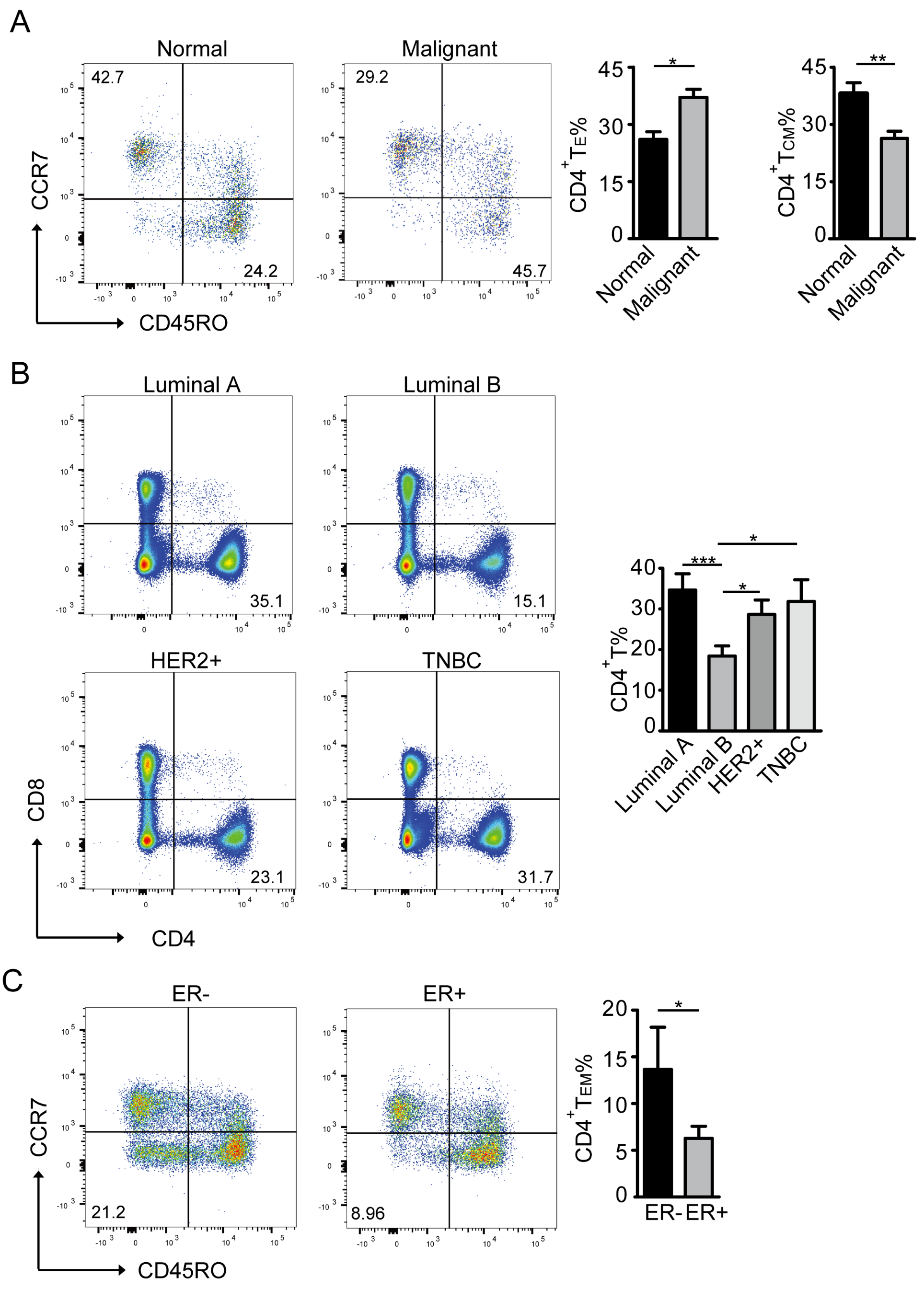

Fig.S1. Distribution of T cell phenotypes in different breast cancer subtypes.

A: The $CD4^+$ T cell subsets in the normal controls(n=19) and breast cancers(n=70). B: The $CD4^+$ T cell percentage in the biologic subtype: Luminal A(n=16), Luminal B(n=26), Her2+(n=20), TNBC(n=6).C: The $CD4^+T_{EM}$ percentage in ER negative(n=17) and ER positive(n=53) groups. The data were presented as mean±SD. *P<0.05; ** P<0.01.

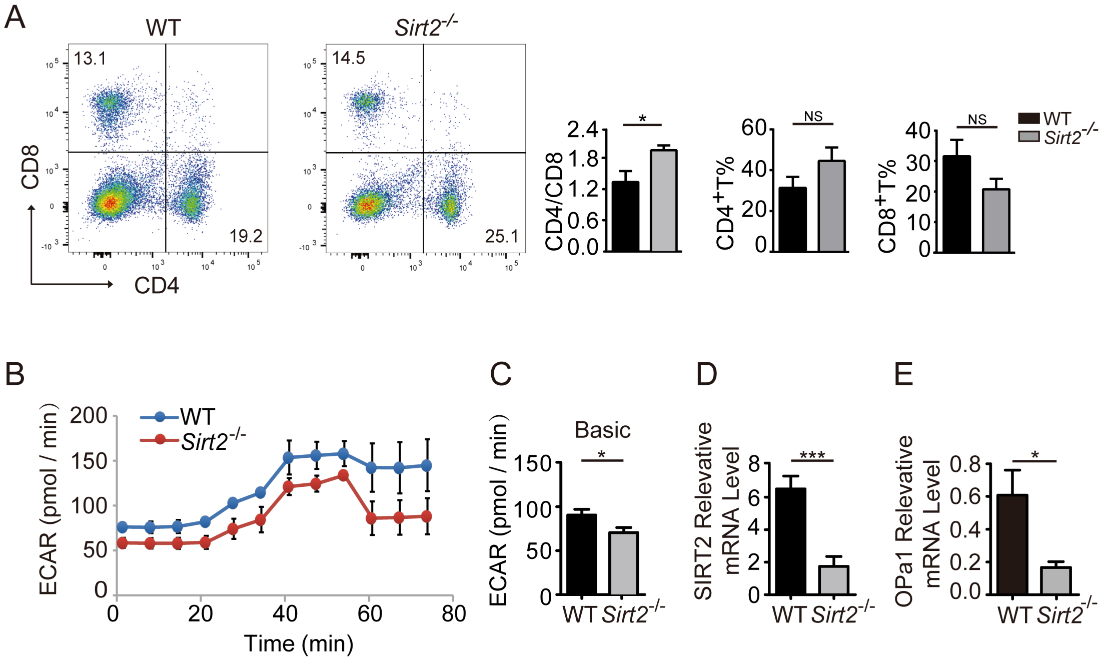

Fig.S2. A: The CD4^{+} and CD8^{+} T percentage in the homologous mouse: wildtype mice (n=11) and *Sirt2*$^{-/-}$(n=9).B.C: ECAR of wild-type(n=6) and *Sirt2*$^{-/-}$ (n= 6) CD8^{+} T cells in real time after the addition of oligomycin,2,4-dinitrophenol(DNP) and retenone. D.E. OPA1 demonstrated a significant decrease in *Sirt2*$^{-/-}$ CD8^{+} T cells. The data were presented as mean±SD. *P<0.05; ** P<0.01; *** P<0.005.
